# Supplementary material for: The interactome of intact mitochondria by cross-linking mass spectrometry provides evidence for coexisting respiratory supercomplexes
Source: Mol Cell Proteomics. 2017 Dec 8;17(2):216–32. doi: 10.1074/mcp.RA117.000470 (PMC5795388; doi:10.1074/mcp.RA117.000470)
Supplement: Supplemental Data [file supp_RA117.000470_133922_0_supp_23978_fzffwf.zip › spectra_annotation/mito_DR_spectra_annotation/63-1-10-1-2-1.pdf]

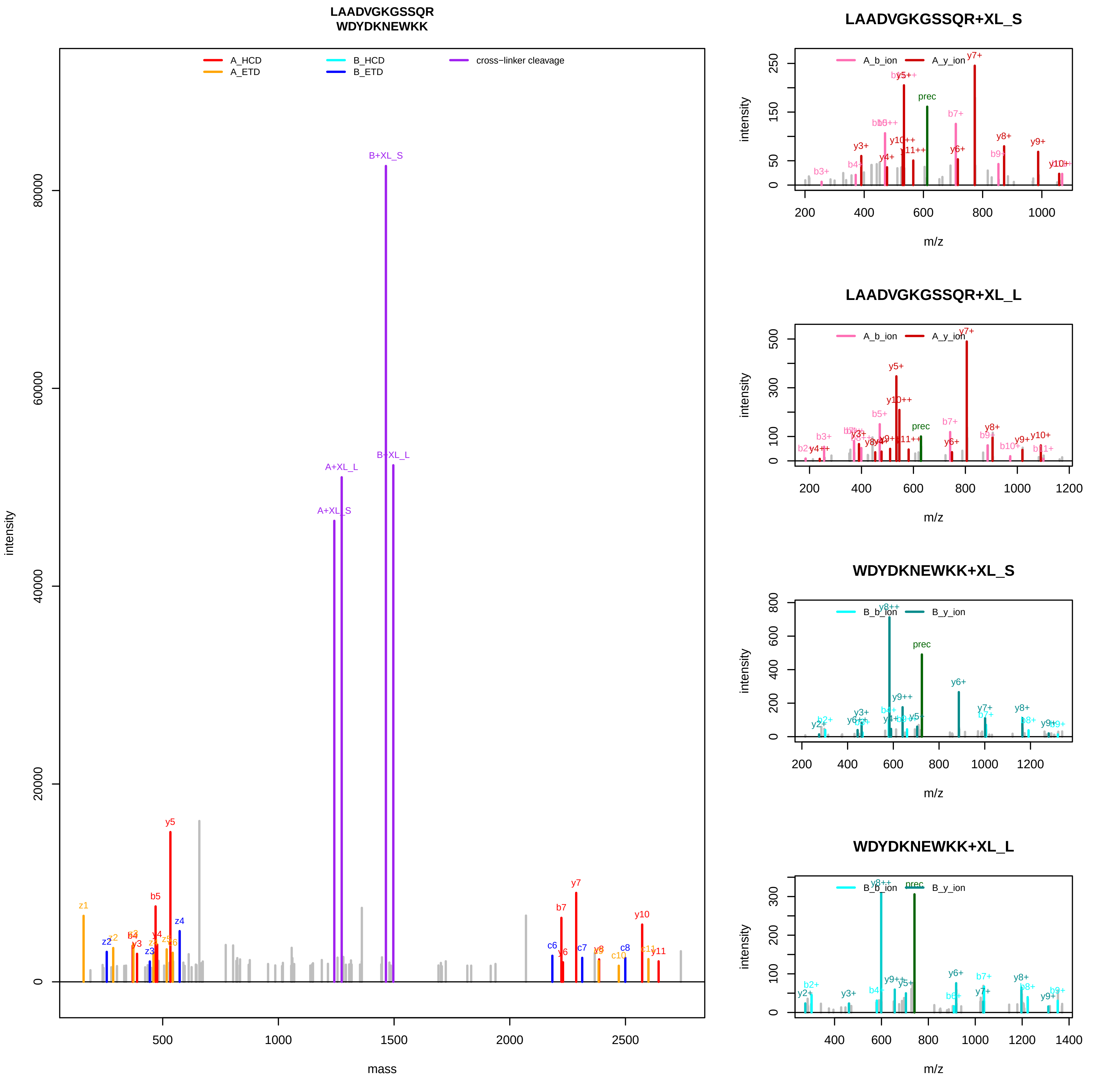

LAADVGKGSSQR
WDYDKNEWKK
A_HCD
A_ETD
B_HCD
B_ETD
cross−linker cleavage
B+XL_S
B+XL_L
A+XL_L
A+XL_S
intensity
mass
0
20000
40000
60000
80000
500
1000
1500
2000
2500
z1
z2
b4
y3
z3
y4
b5
y5
z4
c6
y6
b7
y7
c7
y8
c8
c10
y10
y11
LAADVGKGSSQR+XL_S
LAADVGKGSSQR+XL_L
WDYDKNEWKK+XL_S
WDYDKNEWKK+XL_L
A_b_ion
A_y_ion
B_b_ion
B_y_ion
prec
m/z
y7+
y5+
b7+
y3+
y4+
y6+
y8+
y9+
b9+
b3+
b5+
y10++
y2+
b2+
y9++
y6+
y8++
